# Supplementary material for: The initial melanoma T cell infiltrate is defined by tissue-resident programs restrained by regulatory T cells
Source: Res Sq. 2026 Jul 16:rs.3.rs-8554735. Preprint. [Version 1] doi: 10.21203/rs.3.rs-8554735/v1 (PMC13405490; doi:10.21203/rs.3.rs-8554735/v1)
Supplement: 1 [file NIHPPRS8554735V1-supplement-1.pdf]

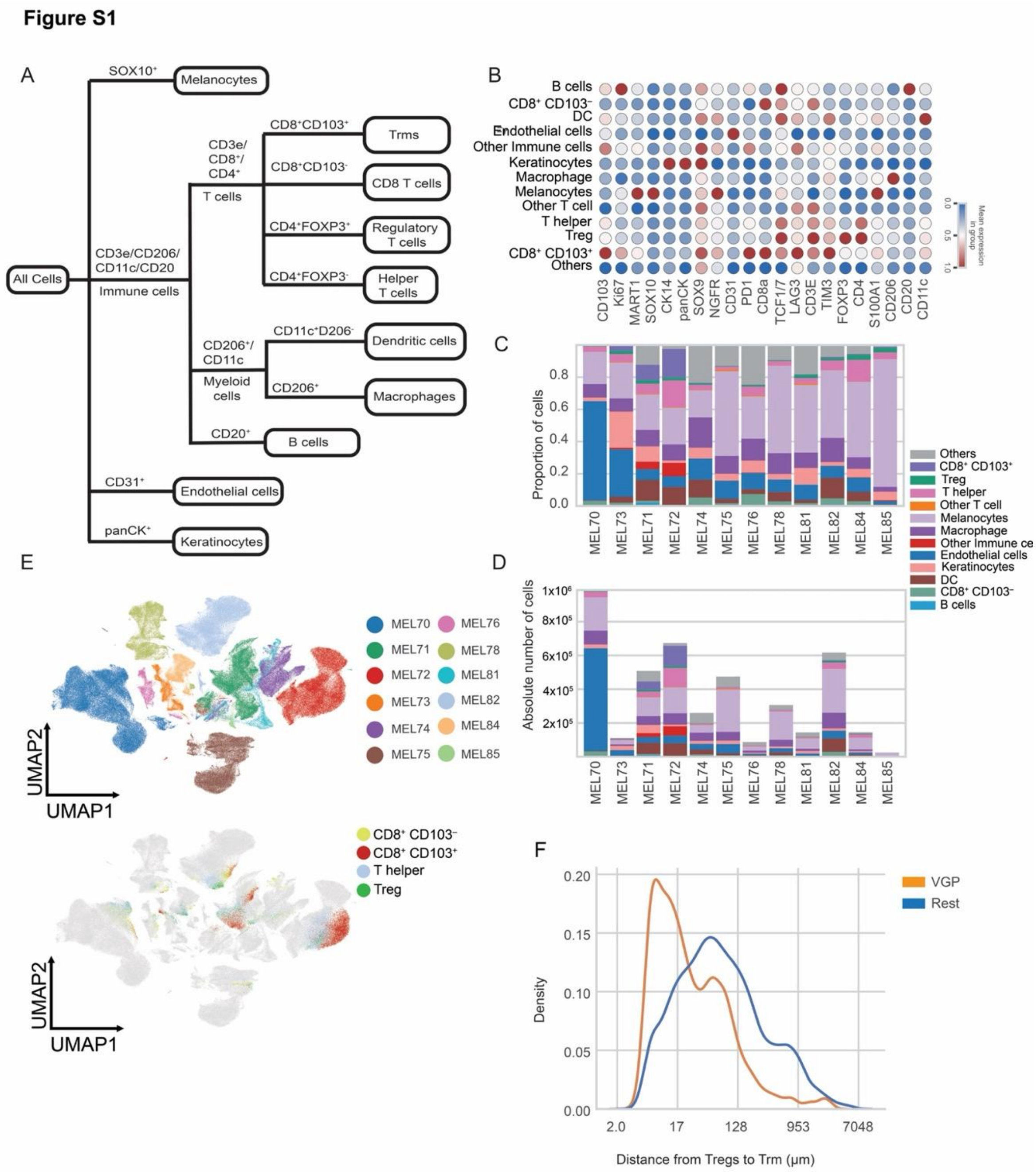

Supplementary Figure S1 | Multiplex imaging panel validation and immune cell composition in human stage II melanoma. **A**, Overview of the 21-marker t-CyCIF antibody panel used to identify immune and stromal cell populations, annotated by lineage. **B**, Heatmap showing scaled (z-score) expression of lineage and functional markers across single-cell clusters identified from multiplex imaging data. CD8$^+$ T cells are stratified by CD103 expression, and CD4$^+$ T cells by Foxp3 expression. **C** and **D**, Relative proportions (**C**) and absolute cell numbers (**D**) of immune, stromal, and melanoma cell populations across all samples. **E**, UMAP plots colored by samples and CD8$^+$CD103$^-$, CD8$^+$CD103$^+$, CD4$^+$Foxp3$^-$, and CD4$^+$Foxp3$^+$ T cell populations. **F**, Distribution plot showing Treg to CD8$^+$CD103$^+$ T cell distances. Significance was determined by a Mann-Whitney U rank test was in **F**.

Figure S2

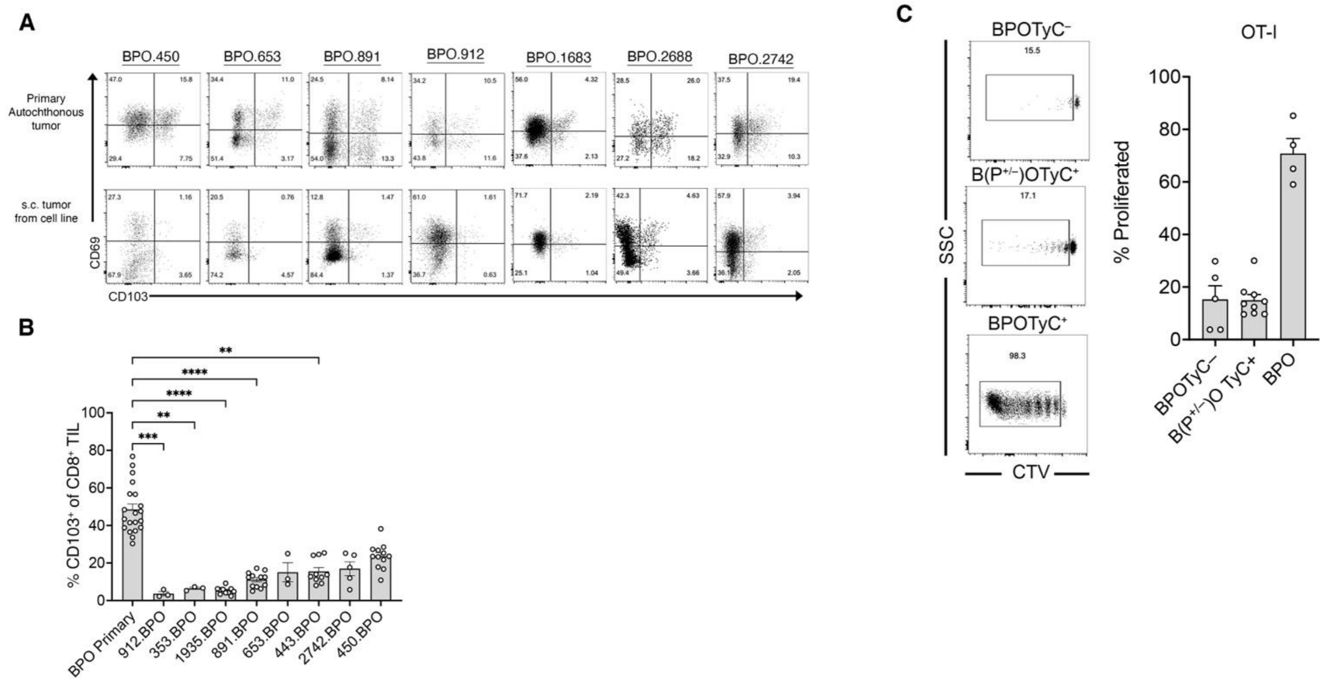

Supplementary Figure S2 | T_{RM}-like $CD8^+$ TIL frequencies in engraftable versus autochthonous melanoma models and OT-I cell proliferation. **A**, Representative flow cytometry plots of CD8 TILs expressing CD103 and CD69 in tumors from BPO-derived cell lines engrafted s.c. and their corresponding primary autochthonous BPO tumors. **B**, Average frequencies of T_{RM}-like $CD8^+$ TILs in primary autochthonous tumors versus s.c. engrafted tumors. BPO primary (autochthonous) tumors were taken at the time of s.c. engraftable experiments and are not the primary BPO-derived cell line tumors. **C**, OT-I proliferation in TdLN after transfer into tumor bearing or tumor-free BPO mice. Graphs display all mice from two (**A** and **B**) or one (**C**) independent experiment. Significance was determined by a Kruskal-Wallis test with Dunn's multiple comparison correction in **B**.

Figure S3

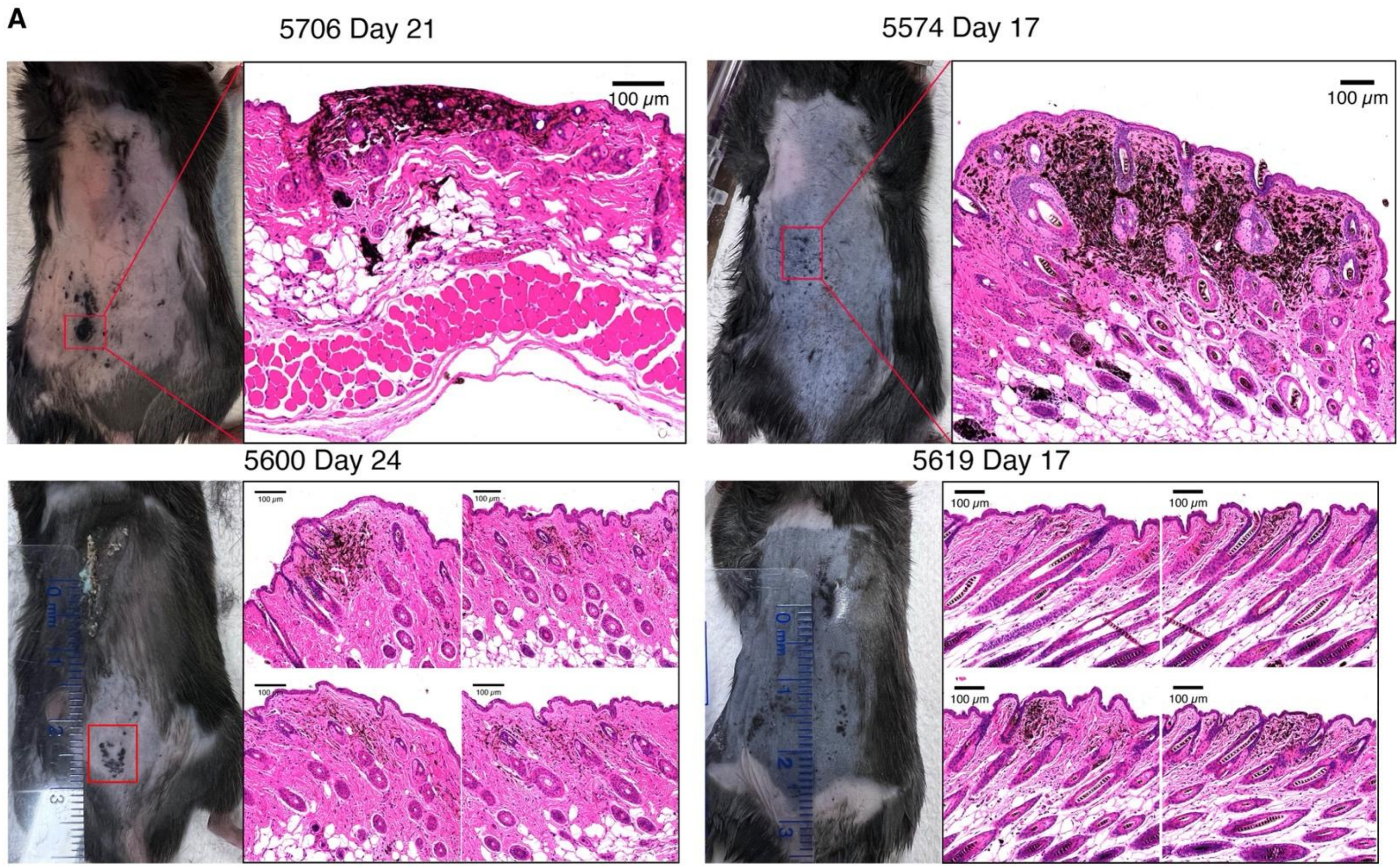

Supplementary Figure S3 | Histopathology of early-stage tumors. A, Representative H&E images showing hyperpigmented lesions confined to upper dermis.

Figure S4

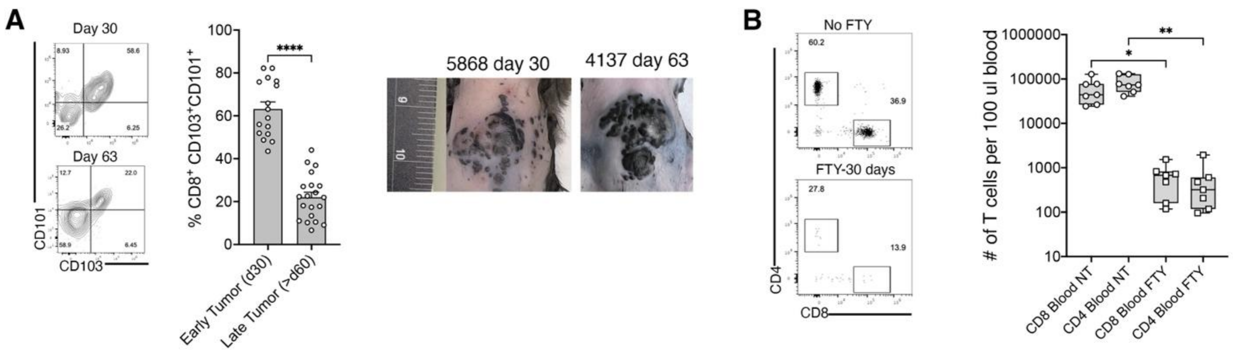

Supplementary Figure S4 | Stability of T_{RM}-like $CD8^+$ TIL niche during tumor progression. A, Representative flow cytometry plots showing CD103 and CD101 expression in early- and late-stage tumors. **B**, Representative flow cytometry plots and cell counts of T cells in peripheral blood after 30 days of FTY720 treatment. Graphs display all mice from 2 independent experiments. Significance was determined by a Mann-Whitney U rank in **A** and a Kruskal-Wallis test with Dunn's multiple comparison correction in **B.**

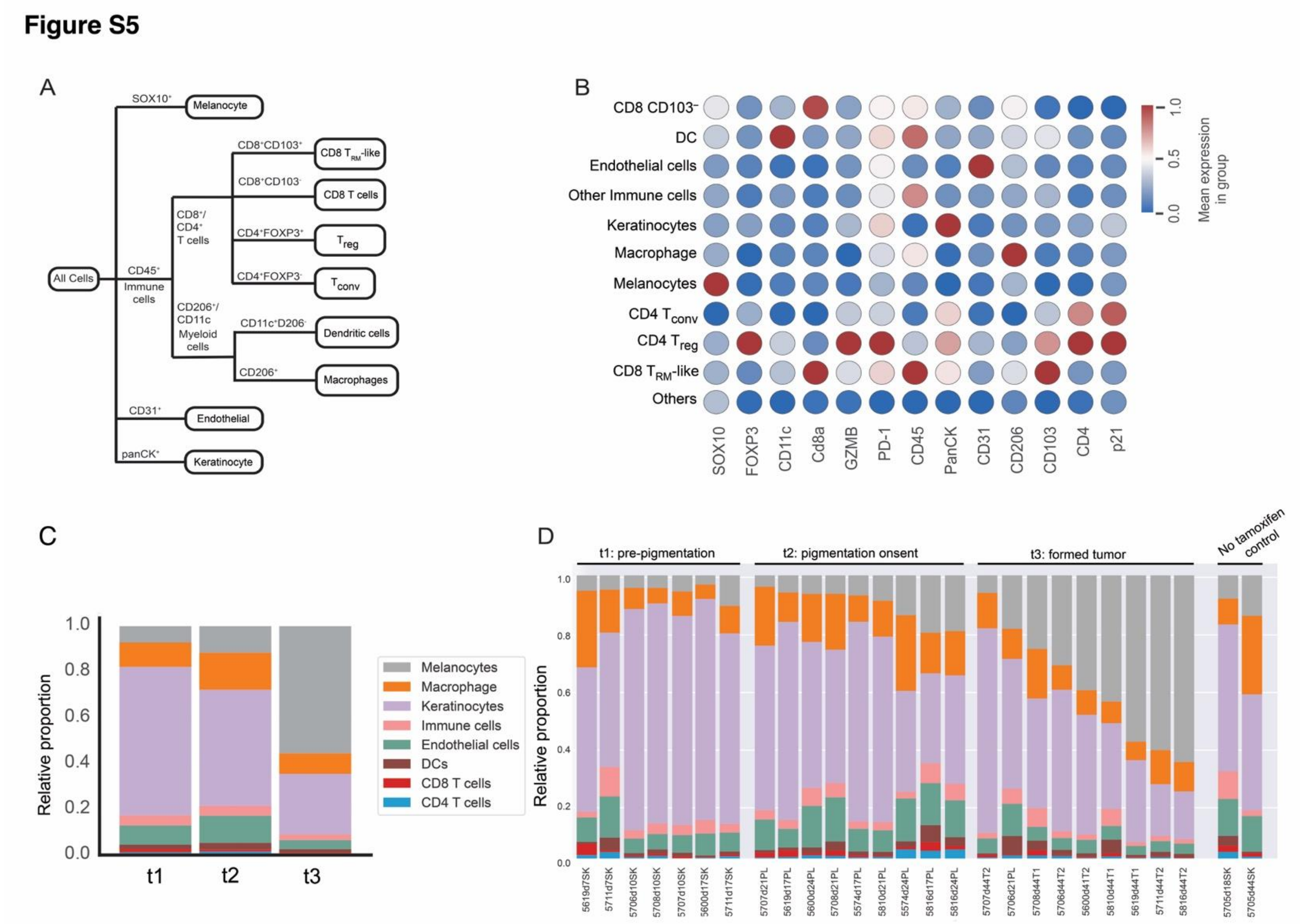

Supplementary Figure S5 | Immune-focused t-CyCIF antibody panel and melanoma biopsy quantification. A, Antibody panel composition for immune cell subset identification in mouse t-CyCIF. **B**, Expression of markers for cell phenotyping. **C** and **D,** Relative proportion of immune cell and stromal cell subsets across time points (**C**) or individual mice (**D**).

Figure S6

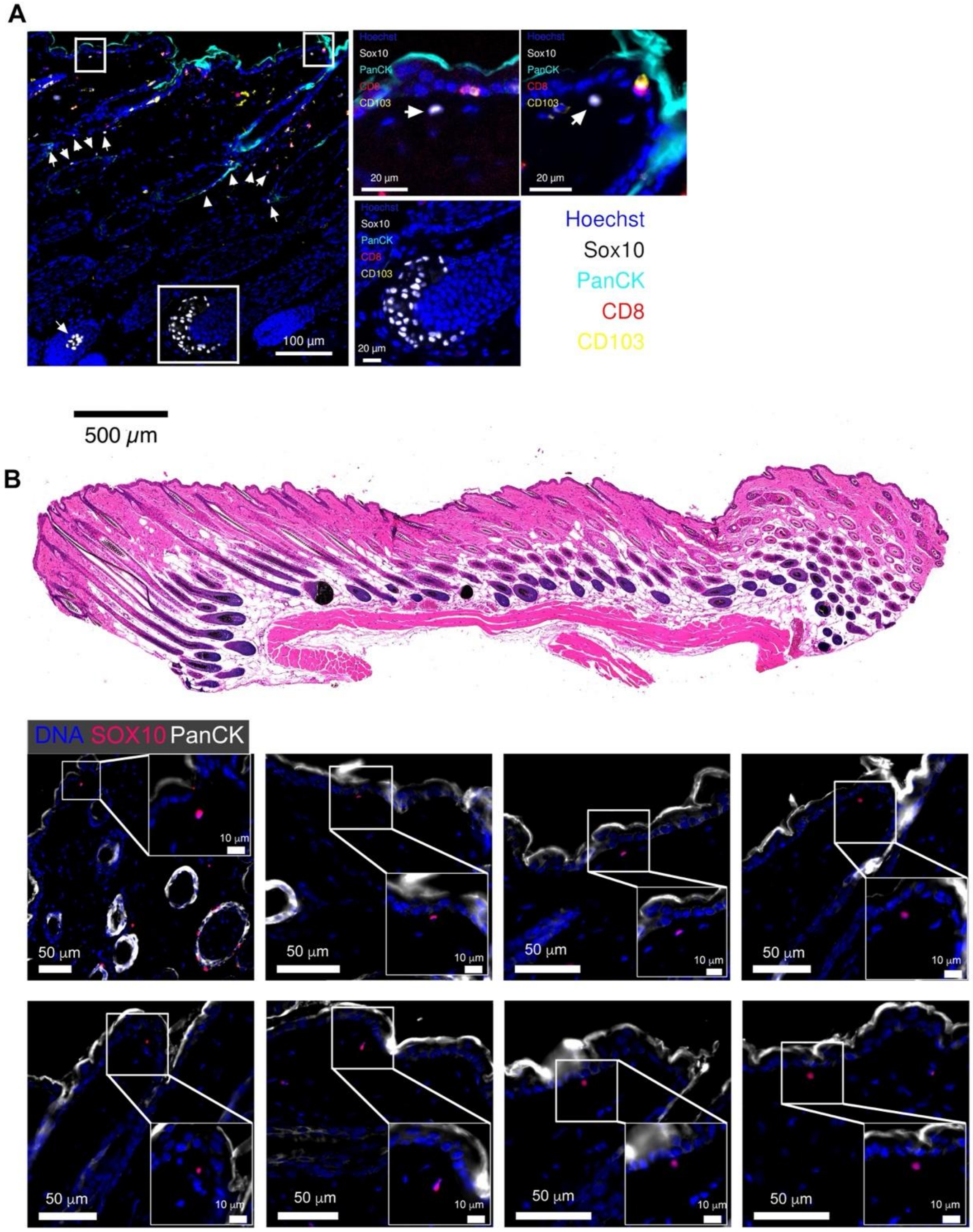

Supplementary Figure S6 | Sox10⁺ melanoma cell localization in pre-pigmentation stage. A, Representative t-CyCIF image showing Sox10⁺ melanoblast localization to lower hair follicle bulb. **B**, Scattered dermal and papillary dermis Sox10⁺ melanoma cells (white arrows) in pre-pigmentation biopsies.

Figure S7

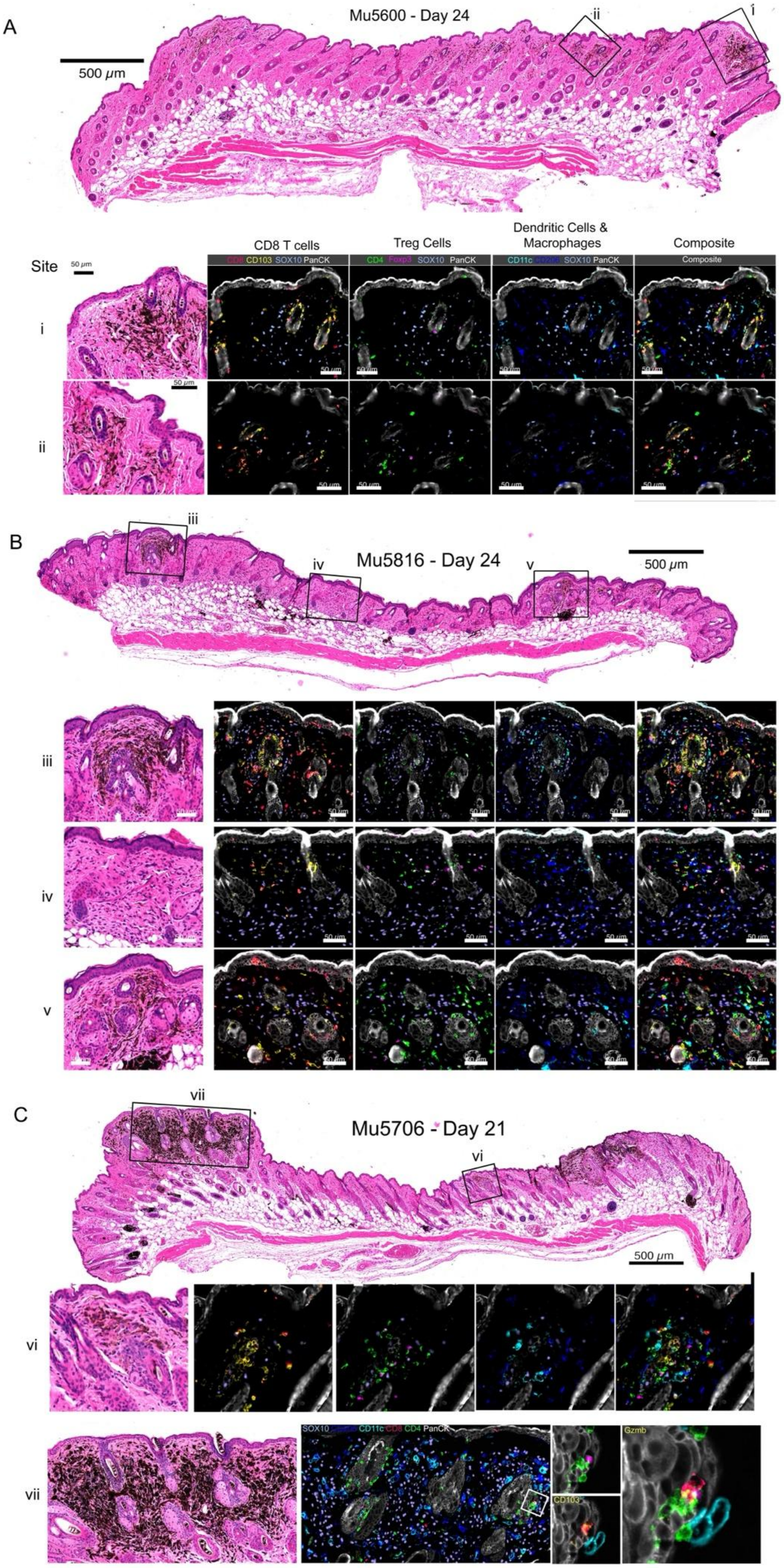
A
Mu5600 - Day 24
500 μm
i
ii
CD8 T cells
Treg Cells
Dendritic Cells & Macrophages
Composite
Site
50 μm
CD8 CD103 SOX10 PanCK
CD4 Foxp3 SOX10 PanCK
CD11c SOX10 PanCK
Composite
B
Mu5816 - Day 24
iii
iv
v
C
Mu5706 - Day 21
vi
vii
SOX10 CD11c CD8 CD4 PanCK
CD103
Gzmb

Supplementary Figure S7 | Heterogeneity of immune microenvironments in early-stage hyperpigmented melanoma cell clusters. A-C, Representative H&E and t-CyCIF images highlighting immune cell composition in hyperpigmented lesions. Sites i-ii, v, and vi show the presence of immune cell clustered with Sox10$^+$ melanoma cells. Clusters with high TRM-like cell density and abundant granzyme B expression. Sites iv-vi exemplify Treg cell enrichment in some hyperpigmented areas. Sites iv and vii, exemplify macrophage-dominant clusters with sparse CD8$^+$ T cells. Inset in site vii shows a single Gzmb$^+$ T_{RM}-like cell in contact with a Treg cell.

Figure S8

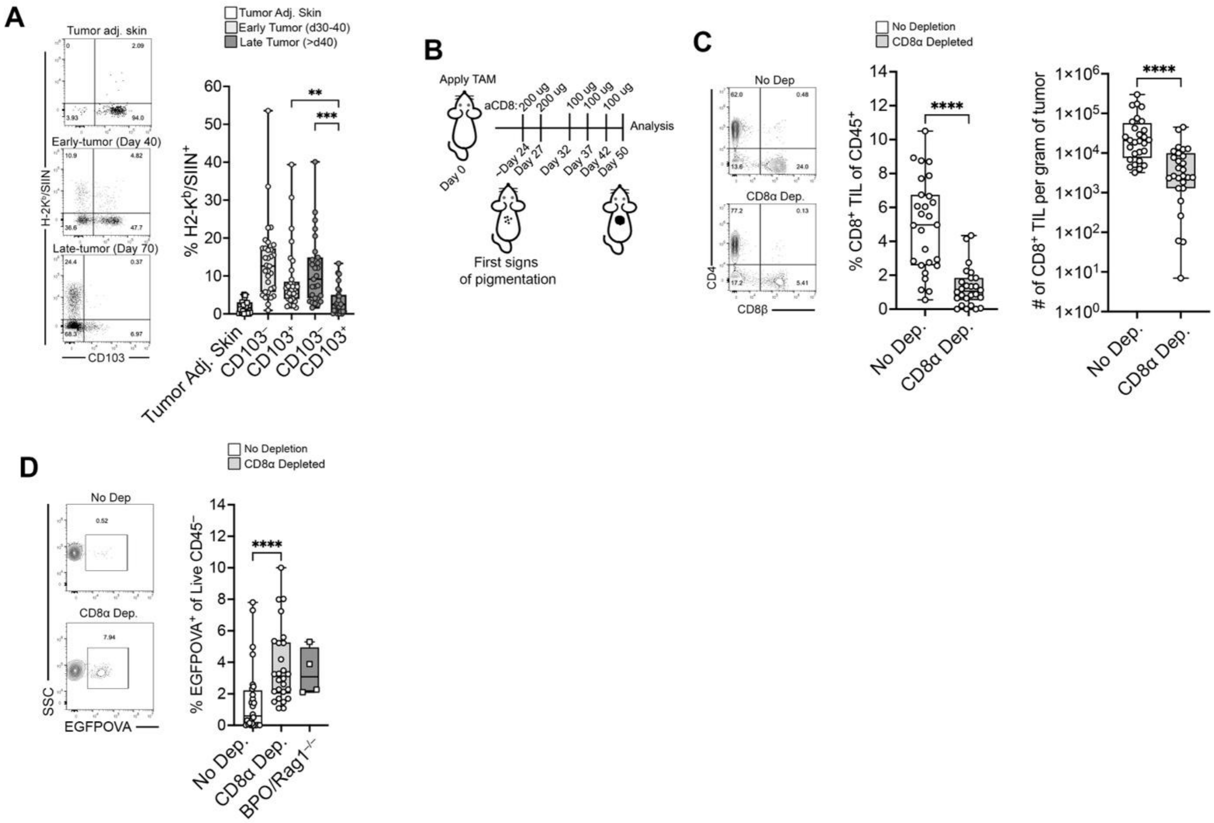

Supplementary Figure S8 | CD8$^+$ T$_{RM}$-like cell tumor specificity and role in immunoediting.
A, H-2K^b/SIINFEKL pentamer staining showing tumor antigen specificity in T$_{RM}$-like versus CD103$^-$ CD8$^+$ TILs at early and late stages of tumor development. **B**, Experimental schematic for CD8$^+$ T cell depletion starting at pigmentation onset.**C**, Confirmation of CD8$^+$ TIL depletion at endpoint. **D**, Frequency of EGFPOVA$^+$ tumor cells in nontreated and CD8-depleted versus immunodeficient (BPO/Rag1$^-$/$^-$) mice. Significance was determined by a Kruskal-Wallis test with Dunn's multiple comparison correction was used in **A** and **D** and a Mann-Whitney U rank test in **C**.

Figure S9

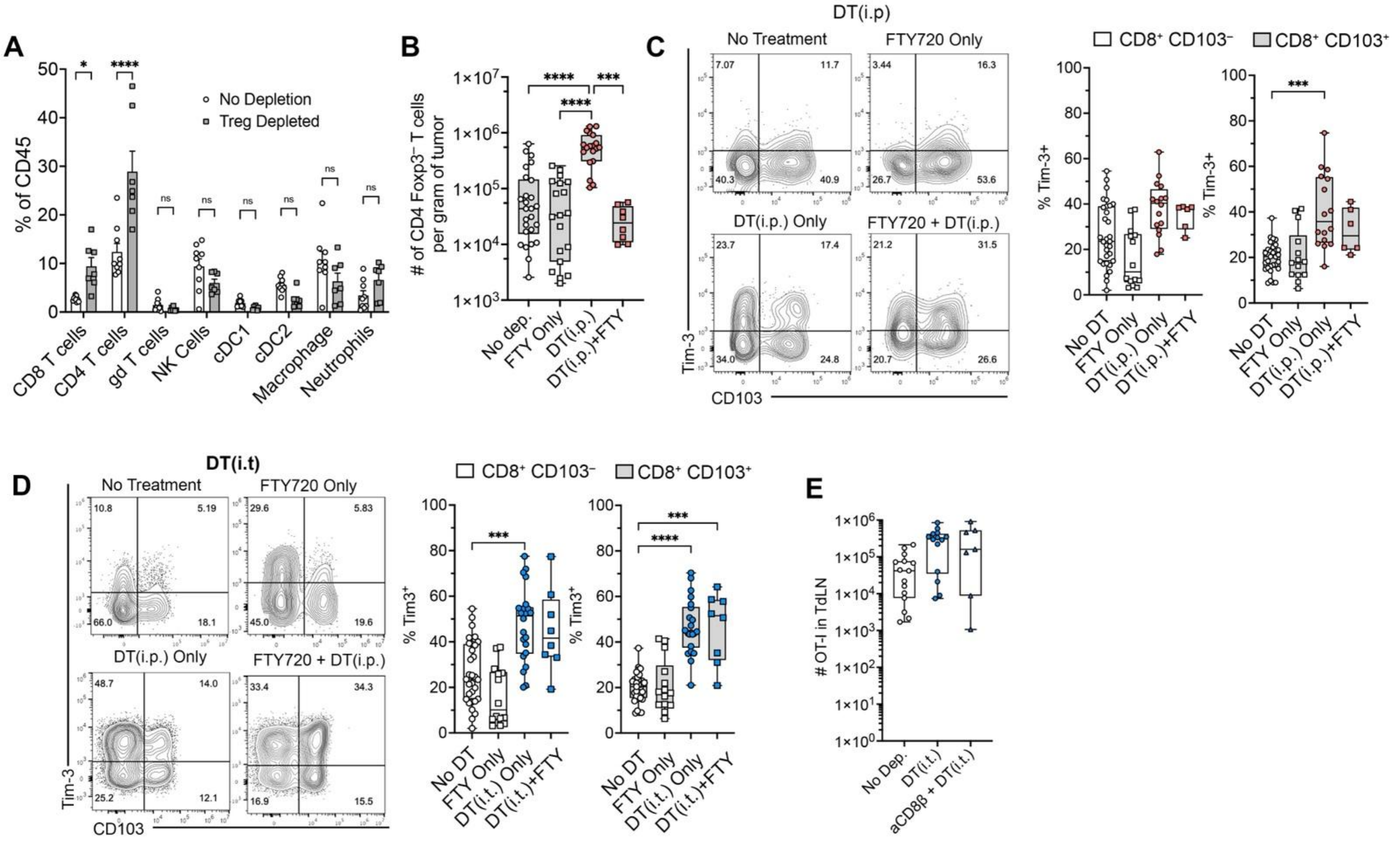

Supplementary Figure S9 | Changes in immune cell composition after Treg depletion. A, Frequencies of CD4⁺ T cells, γδ T cells, dendritic cells, macrophages, NK cells, and neutrophils after systemic Treg depletion. **B**, Recruitment of Foxp3⁻ CD4⁺ Tconv cells after systemic Treg depletion ± FTY720. **C**, Tim-3 expression on CD8⁺ TILs after systemic Treg depletion ± FTY720. **D**, Tim-3 expression on CD8⁺ TIL subsets after local (DT i.t.) Treg depletion. **E**, OT-I T cell counts in TdLN after Treg depletion with prior anti-CD8β antibody treatment. Graphs display all mice from 2-3 independent experiments. Significance was determined by a Kruskal-Wallis test with Dunn's multiple comparison correction in **A-D**.

Supplemental Figure 10

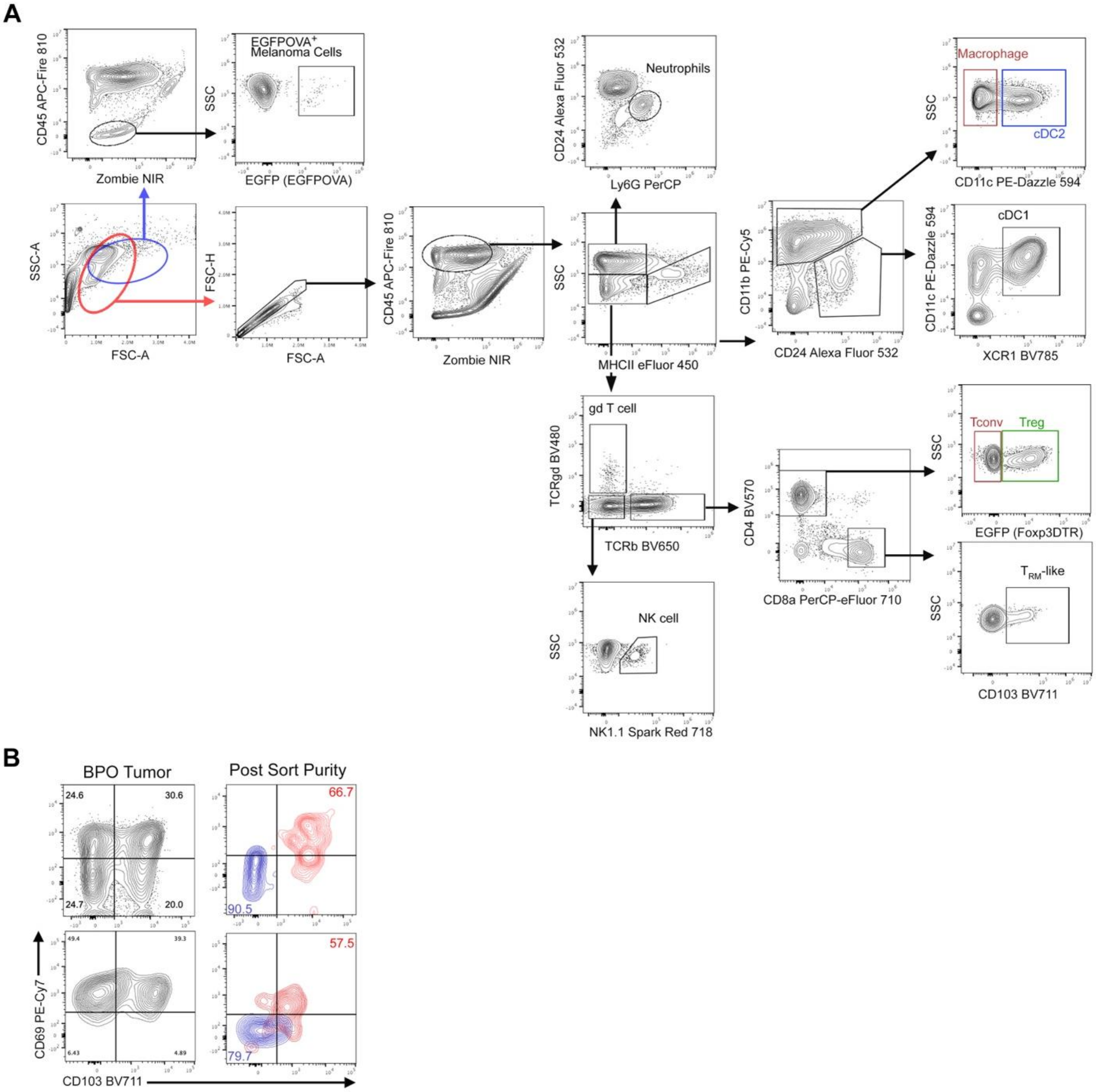

Supplementary Figure S10 | Flow cytometry gating strategy. A, Separate gates were used to isolate lymphocytes and tumor cells. Tumor cells were identified to be within the CD45⁻ SSChi fraction. Cell populations were identified as follows; Neutrophils: CD45⁺ SSChi MHCII⁻ CD24int Ly6G⁺, Macrophage: CD45⁺ MHCII⁺ CD11b⁺ CD24⁻ CD11c⁻, cDC1: CD45⁺ MHCII⁺ CD24⁺ CD11b⁻ CD11c⁺ XCR1⁺, gd T cell: CD45+ SSClow TCRb⁻ TCRgd⁺, NK cell: CD45⁺ SSClow TCRb⁻ TCRgd⁻ NK1.1⁺, CD8 T cell: CD45⁺ SSClow TCRb⁺ CD8a⁺, CD4 T cell: CD45⁺ SSClow TCRb⁺ CD4⁺. Treg cells were identified as CD4⁺ T cell with Foxp3EGFPDTR⁺. TRM-like cells were identified as CD8⁺ T cell with CD103 expression. **B**, Mid-sort analysis for bulk RNASeq experiment. Cell purity for two samples was determined during the sort to confirm purity.
